# Fracture and Intravascular Retention of a Radial Arterial Catheter Following Reinsertion of the Introducer Needle: Point‐of‐Care Ultrasonography‐Guided Localization and Surgical Retrieval

**DOI:** 10.1002/ccr3.71515

**Published:** 2025-11-20

**Authors:** Takuya Shiraishi, Masashi Yoshida

**Affiliations:** ^1^ Department of Anaesthesiology Hitachi General Hospital Hitachi Ibaraki Japan

**Keywords:** arterial catheterization, catheter fracture, patient safety, point‐of‐care ultrasonography, radial artery

## Abstract

Reinserting an introducer needle into a radial arterial catheter can shear the cannula and leave an intravascular fragment. When bleeding from the shaft or resistance occurs, manipulation should be stopped, fracture should be suspected, and point‐of‐care ultrasonography (POCUS) should be used to localize the fragment and expedite safe surgical retrieval.

## Graphical abstract

Point‐of‐care ultrasonography enabled rapid localization of the fractured intravascular catheter fragment and guided safe surgical retrieval.

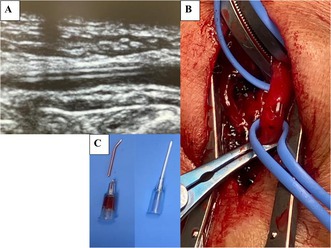

## 1 Introduction

Continuous arterial pressure monitoring via radial cannulation is ubiquitous in peri‐operative and critical care settings. Although overall complication rates are low, device‐related events can be consequential. Among these, catheter fracture with intravascular retention is exceptionally rare yet clinically important because retained fragments may embolise or compromise distal perfusion, necessitating urgent identification and removal. Large series underline the rarity of serious adverse events associated with radial artery cannulation. In a review of 19,617 cannulations, major complications were reported in fewer than 1%, and in a contemporary cohort of 62,626 arterial lines, severe vascular or nerve events occurred in 3.4 per 10,000 placements [1, 2]. In both the Seldinger and over‐the‐needle approaches, mechanical notching, kinking, or shear can occur if resistance is encountered or if handling is suboptimal, particularly at the needle–catheter interface.

We report a case in which oozing at the insertion site prompted suspicion of device damage and an attempt to remove the catheter. Resistance was encountered, and after forceful withdrawal, the missing distal tip was discovered with intravascular retention. Immediate point‐of‐care ultrasonography (POCUS) localized the fragment and enabled planned open retrieval. We synthesize practical lessons on when to stop and reassess, how to triage imaging and retrieval, and which preventive steps may reduce risk in routine practice. This case is relevant to everyday anesthetic workflows because a commonly used 22‐gauge radial catheter was placed during routine set‐up, conditions under which many clinicians may plausibly encounter similar early warning signs.

## 2 Case History/Examination

A 68‐year‐old man (168 cm, 64 kg) with hypertension and dyslipidaemia was scheduled for a laparoscopic distal pancreatectomy. Preprocedural vascular assessment (e.g., Allen/Barbeau test) was unremarkable. Intraoperative monitoring included a left radial arterial line. A 22‐gauge SURFLO Flash catheter (Terumo, Tokyo, Japan) was used.

Cannulation was performed by palpation without the use of ultrasound or guidewires. The first puncture was unsuccessful, and the second puncture achieved arterial access; however, as the needle was advanced, the backflow of arterial blood into the inner needle ceased, suggesting that the needle had passed through the vessel. The introducer needle was completely withdrawn, and the catheter was slowly retracted by several millimeters until brisk arterial backflow was observed. At that point, the same needle was carefully reinserted into the indwelling cannula to provide stiffness for advancing the catheter into the arterial lumen. This maneuver represents a local modification of the over‐the‐needle technique routinely practiced at our institution, where commercially available guidewire‐equipped arterial cannulation kits are not used.

Mild resistance was felt during reinsertion; however, after several careful attempts, the needle appeared to align properly with the catheter, and the catheter was successfully advanced. A pulsatile arterial waveform was confirmed on the monitor after connection of the line.

Although preparing to secure the catheter, continuous oozing was noted from the catheter shaft rather than from the skin puncture site, raising suspicion of structural damage. Removal was attempted, but resistance was encountered, and after forceful withdrawal, the distal tip of the catheter was found to be missing. On examination, the radial pulse remained palpable distal to the puncture site, and the hand was warm with normal capillary refill.

## 3 Differential Diagnosis, Investigations and Treatment

### 3.1 Differential Diagnosis

Bedside considerations included: (i) cannula fracture with intravascular retention, (ii) partial transection or side‐hole formation of the catheter, (iii) arterial wall injury (intimal flap, dissection) with oozing, (iv) through‐and‐through puncture with extravasation/haematoma, and (v) early pseudoaneurysm formation.

### 3.2 Investigations

**FIGURE 1:**
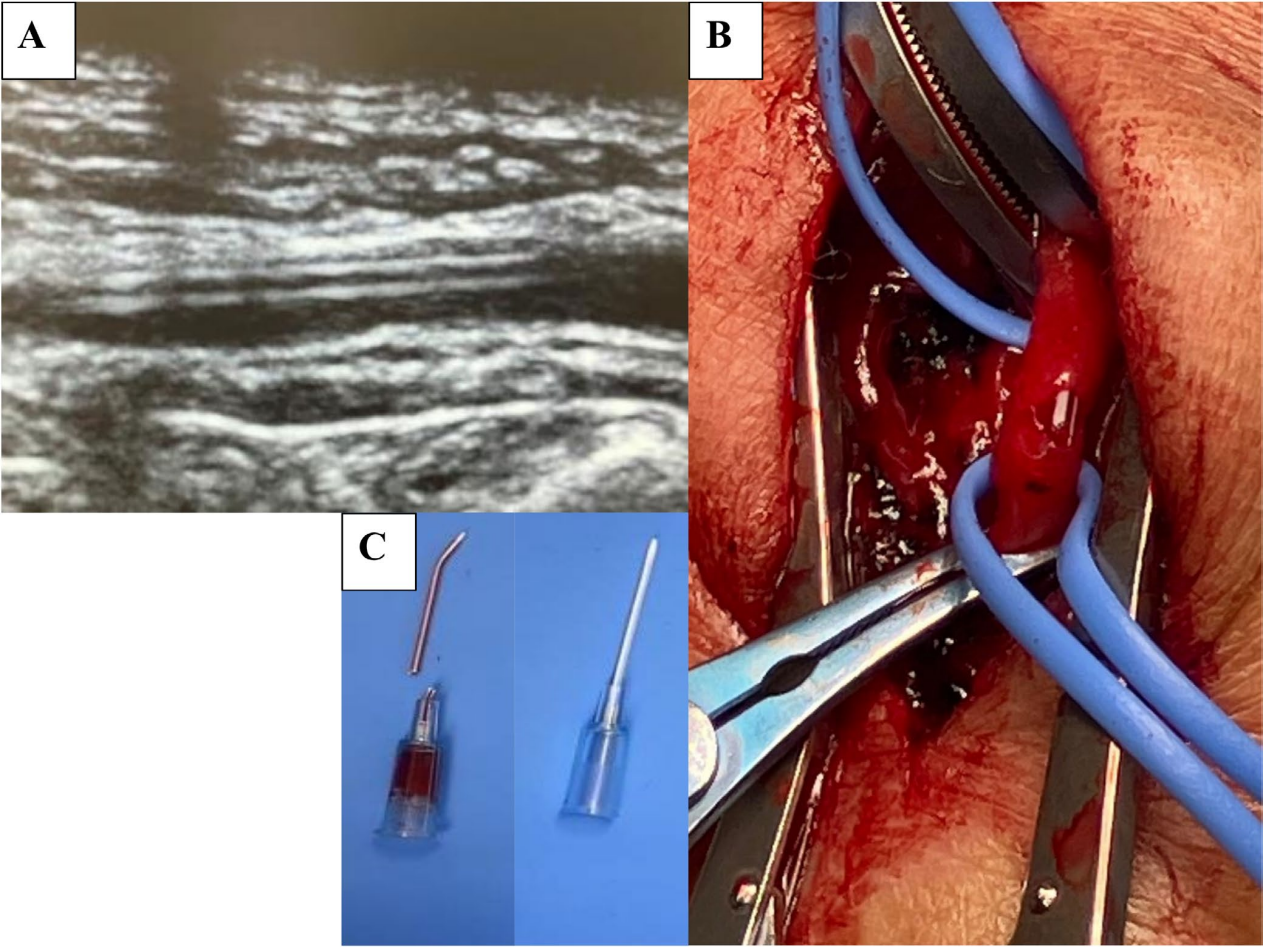
(A) Point‐of‐care ultrasound (short‐axis view) of the left radial artery demonstrating a linear echogenic intraluminal fragment (arrowheads), consistent with a fractured catheter tip. (B) Intraoperative photograph of open retrieval performed by the cardiovascular surgery team showing vascular exposure and control with vessel loops. (C) Retrieved fractured arterial catheter on the left, shown alongside an intact catheter on the right for comparison.

Immediate POCUS of the left radial artery revealed a linear echogenic intraluminal fragment measuring approximately 20 mm with preserved distal perfusion and no flow‐limiting thrombus (Figure 1A). The fragment was superficial at the wrist level, consistent with a fractured catheter tip.

### 3.3 Treatment

After multidisciplinary discussion with vascular surgery, open retrieval with arterial repair was performed without complications (Figure 1B). The retrieved fractured arterial catheter is shown alongside an intact catheter (Figure 1C). For uninterrupted monitoring, a new arterial line was placed in the contralateral radial artery before proceeding with the planned operation.

## 4 Conclusion and Results (Outcome and Follow‐Up)

The laparoscopic procedure was performed as scheduled. The patient's postoperative course was uneventful; there were no ischaemic or neurological complications, and he was discharged on postoperative day 7 in good condition. At follow‐up, the patient reported no ischaemic symptoms and expressed satisfaction with the recovery and outcome.

## 5 Discussion

This case illustrates how an uncommon but clinically relevant device complication can be recognized and managed within a routine peri‐operative workflow. A large number of studies underscore the rarity of serious events. In a classic review of 19,617 radial cannulations, major complications occurred in fewer than 1%, with temporary radial occlusion of approximately 19.7%, serious ischaemic damage of approximately 0.09%, pseudoaneurysm of approximately 0.09%, and sepsis of approximately 0.13%. Overall, radial artery cannulation is a safe procedure [1]. In more contemporary data from 62,626 arterial lines at a single centre, severe vascular or nerve complications occurred in 3.4 per 10,000 (0.034%), with higher rates for larger‐bore catheters, and the radial site accounted for 94.5% of placements [2].

Catheter fracture with intravascular retention is rare, yet prompt recognition is essential to avoid embolization and limb‐threatening sequelae. In our patient, bleeding that appeared to originate from the catheter shaft rather than the skin provided a simple bedside clue to structural compromise. The sequence of events—an initial failed puncture followed by a second puncture that likely resulted in arterial transfixion, subsequent complete withdrawal and reinsertion of the introducer needle after confirming arterial backflow, onset of shaft bleeding, and eventual discovery of a missing tip—supports a mechanism in which the catheter wall was notched at the needle–catheter interface during reinsertion of the introducer needle, and subsequently sheared during manipulation or withdrawal, leading to an oblique distal fracture. Factors that plausibly increased susceptibility include the use of a small‐bore catheter without a safety mechanism and reinsertion of the introducer needle under slight resistance, both of which can magnify the consequences of subtle misalignment between the needle and catheter.

Once suspected, ultrasound serves as the first‐line tool to confirm the diagnosis, define the fragment's location, and document distal patency, thereby enabling a deliberate retrieval plan that minimizes delay to definitive care—a pattern mirrored in recent reports where POCUS was pivotal in localizing retained radial fragments before operative removal and, in some cases, documenting proximal migration [3, 4]. POCUS also facilitates documentation for multidisciplinary decision‐making and counseling, should retrieval be deferred.

Published case reports further clarify that most fractures are not produced during initial cannulation but rather by accidental transection during dressing removal or catheter removal, or by patient self‐discontinuation; examples include transection at removal, transection while cutting dressings or sutures, and self‐extraction with subsequent retention [5, 6, 7]. Beyond the radial artery, brachial‐artery catheter fragments have been reported, with retrieval tailored to fragment depth and local expertise, supporting a spectrum from open exploration to endovascular solutions [8]. Against this background, reports explicitly implicating structural damage at the needle–catheter interface during reinsertion of the introducer needle after complete withdrawal remain scarce [9].

Our case illustrates this plausible mechanism and emphasizes a series of preventive lessons: any resistance encountered during needle withdrawal, reinsertion, or catheter advancement should prompt immediate cessation and reassessment; forceful reinsertion under resistance must be avoided, as even minor misalignment at the interface can predispose the device to shear.

In addition, adopting guidewire‐assisted or safety‐engineered arterial cannulation kits, using ultrasound guidance more liberally after multiple punctures or when tracking is uncertain, and reconsidering the technique of complete needle withdrawal and reinsertion itself may all reduce the risk of similar complications in routine practice. Embedding a concise “stop–ultrasound–decide–retrieve” reflex into daily workflow may further shorten the time to definitive management when subtle warning signs arise.

This report has several limitations. This was a single‐patient case; therefore, causality and generalisability are inherently limited. The proposed mechanism of needle–catheter interface notching and shear is inferential based on the observed sequence and device inspection rather than direct visualization. Our management pathway, including ultrasound‐first localization and open retrieval, reflects local expertise and device availability and may not be universally applicable. Further data from comparative series are needed to define the incidence, modifiable risk factors, and relative merits of open versus endovascular retrieval.

## 6 Patient/Carer Perspective

From the patient's perspective, early acknowledgement and a clear explanation of the complication, together with ultrasound confirmation and planned retrieval, were reassuring. The patient reported no ischaemic symptoms after surgery and expressed satisfaction with the recovery and outcome. The ability to proceed with the planned operation after contralateral arterial access was also considered.

## Author Contributions

**Takuya Shiraishi:** conceptualization, investigation, supervision, visualization, writing – original draft, writing – review and editing. **Masashi Yoshida:** investigation, resources, writing – review and editing.

## Funding

The authors have nothing to report.

## Disclosure

The authors have nothing to report.

## Ethics Statement

Ethical approval was not required for this single‐patient case report according to our institutional policy; written informed consent for publication was obtained from the patient.

## Consent

Written informed consent for the publication of this case, images, and de‐identified data was obtained from the patient in accordance with journal policy. The documentation is available upon request.

## Conflicts of Interest

The authors declare no conflicts of interest.

## Data Availability

De‐identified data related to this case are available from the corresponding author upon reasonable request.

## References

[1] B. Scheer , A. Perel , and U. J. Pfeiffer , “Clinical Review: Complications and Risk Factors of Peripheral Arterial Catheters Used for Haemodynamic Monitoring in Anaesthesia and Intensive Care Medicine,” Critical Care 6, no. 3 (2002): 199–204. 12133178 10.1186/cc1489 PMC137445

[2] G. Nuttall , J. Burckhardt , A. Hadley , et al., “Surgical and Patient Risk Factors for Severe Arterial Line Complications in Adults,” Anesthesiology 124, no. 3 (2016): 590–597. 26640979 10.1097/ALN.0000000000000967

[3] A. Byskosh and K. Olsen , “Retained Arterial Catheter Following Patient Self‐Discontinuation—A Case Report,” Journal of Surgical Case Reports 1 (2024): rjad727. 10.1093/jscr/rjad727 PMC10795922 38239372

[4] A. I. Smith , “Retained Radial Artery Catheter Fragment,” Oxford Medical Case Reports 6 (2024): 229–231. 10.1093/omcr/omae053 PMC11162587 38860014

[5] L. Tollinche , J. Jackson , M. La , D. Desiderio , and C. Yeoh , “Case Report: Transection of Radial Arterial Catheter Requiring Surgical Intervention,” Journal of Intensive and Critical Care 4, no. 1 (2018): 3. 29780973 PMC5954833

[6] U. I. Hamid , A. Collins , C. McConkey , and P. Sidhu , “Accidental Transection of a Radial Artery Cannula,” BMJ Case Reports 2012 (2012): bcr2012006753. 10.1136/bcr-2012-006753 PMC4544230 22949001

[7] S. K. Moon , J. C. Gong , J. H. Kim , et al., “A Retained Catheter Fragment in Radial Artery Caused by Accidental Catheter Transection During Arterial Catheter Removal,” Journal of Anesthesia 26, no. 4 (2012): 625–626. 22484914 10.1007/s00540-012-1388-4

[8] Y. Y. Chen , “SonographyGuided Endovascular Retrieval of Fracture‐d Angiocatheter in Brachial Artery: A Case Report,” Vascular and Endovascular Surgery 59, no. 4 (2025): 424–427. 39568294 10.1177/15385744241302554

[9] S. Y. Lee , H. S. Na , M. H. Kim , et al., “A Sheared Catheter Fragment in the Wrist After Arterial Cannulation Attempt: A Case Report,” Korean Journal of Critical Care Medicine 25, no. 2 (2010): 118–121.

